# Convergent evolution of coloration in experimental introductions of the guppy (*Poecilia reticulata*)

**DOI:** 10.1002/ece3.4418

**Published:** 2018-08-15

**Authors:** Cynthia Dick, Jasmine Hinh, Cheryl Y. Hayashi, David N. Reznick

**Affiliations:** ^1^ Department of Evolution, Ecology and Organismal Biology University of California‐Riverside Riverside California; ^2^ Division of Invertebrate Zoology and Sackler Institute for Comparative Genomics American Museum of Natural History New York New York

**Keywords:** coloration, convergent evolution, experimental introduction, guppy, RNA‐seq

## Abstract

Despite the multitude of examples of evolution in action, relatively fewer studies have taken a replicated approach to understand the repeatability of evolution. Here, we examine the convergent evolution of adaptive coloration in experimental introductions of guppies from a high‐predation (HP) environment into four low‐predation (LP) environments. LP introductions were replicated across 2 years and in two different forest canopy cover types. We take a complementary approach by examining both phenotypes and genetics. For phenotypes, we categorize the whole color pattern on the tail fin of male guppies and analyze evolution using a correspondence analysis. We find that coloration in the introduction sites diverged from the founding Guanapo HP site. Sites group together based on canopy cover, indicating convergence in response to light environment. However, the axis that explains the most variation indicates a lack of convergence. Therefore, evolution may proceed along similar phenotypic trajectories, but still maintain unique variation within sites. For the genetics underlying the divergent phenotypes, we examine expression levels of color genes. We find no evidence for differential expression, indicating that the genetic basis for the color changes remains undetermined.

## 1 INTRODUCTION

Examples of microevolution in populations have demonstrated that evolution can proceed much more rapidly than previously believed (Darwin, 1859; Hendry & Kinnison, 1999). Species introductions provide one way to examine evolution in action as populations experience new environments, resources, competitors, or predators (Reznick & Ghalambor, 2001). Examples include *Drosophila* (fruit fly) body size clines when adapting to a new climate and *Poecilia* (guppy) life history evolution in response to altered predator community (Huey, Gilchrist, Carlson, Berrigan, & Serra, 2000; Reznick & Bryga, 1987; Reznick, Bryga, & Endler, 1990).

When introductions are replicated, they can provide information about the repeatability of rapid evolution. The populations in question can respond to the selection pressures by evolving convergently along similar trajectories. Alternatively, different populations may adapt to the same selection by evolving along different trajectories (historical contingency) or may instead respond via phenotypic plasticity. Convergent evolution has been proposed as evidence that the options for adaptive evolution are limited (Achaz, Rodriguez‐Verdugo, Gaut, & Tenaillon, 2014). Historical contingency occurs when random events unique to each population in question cause them to evolve along different trajectories (Blount, Borland, & Lenski, 2008; Gould, 2002). Plasticity could cause divergence among populations if differences are induced as a response to unique features of the environment (Oke et al., 2016).

Trinidadian guppies provide the opportunity to evaluate whether populations in replicated introductions evolve convergently. Guppies from downstream communities co‐occur with many predators, hence are referred to as high‐predation (HP) communities. Guppies from headwater streams above barrier waterfalls live with few other species and experience reduced predation, hence are referred to as low‐predation (LP) communities. HP guppies differ from LP guppies in a diversity of attributes, including life history, morphology, and behavior (Endler, 1995; Reznick & Bryga, 1996; Reznick & Endler, 1982). HP guppies have occasionally crossed barrier waterfalls to colonize LP sites (Willing et al., 2010), after which these guppies evolve and acquire LP phenotypes. HP guppies have been introduced into previously guppy‐free LP environments multiple times to examine evolution in action. After the introduction, fish have evolved traits similar to natural LP guppies (Endler, 1980; Reznick, Shaw, Rodd, & Shaw, 1997; Reznick et al., 1990).

Guppy male ornamental coloration is thought to evolve under convergent evolution between HP and LP populations. Although ornamental coloration is a complex multicomponent trait, LP males are known to have greater spot size, area, and number than HP males (Endler, 1995). This is caused by a trade‐off between natural and sexual selection: natural selection favors less colorful males, while sexual selection in the form of female choice favors more colorful males (Endler, 1983).

Researchers have conducted several introductions in the Caroni drainage system to examine color evolution. Kemp, Reznick, Grether, and Endler (2009) re‐examined the original Aripo introduction by Endler (1980) as well as the El Cedro introduction to determine whether body color evolution was predictable. They found that body color did increase in both introductions, but along different routes. Orange/yellow, black, and iridescence increased in Aripo, but only iridescence increased in El Cedro and orange and black actually declined. More recent introductions in 2008 and 2009 have been made from Guanapo HP into the LP Upper Lalaja, Lower Lalaja, Taylor, and Caigual Rivers. Gordon et al.'s work examined color evolution in two of those introductions. They found that black coloration decreased in Upper and Lower Lalaja compared to Guanapo HP, while orange/yellow coloration increased (Gordon et al., 2015). Common garden experiments indicated that black changes were most likely due to plasticity and orange/yellow changes were the result of genetic effects. Kemp, Batistic, and Reznick (in review) recently determined that Upper Lalaja, Lower Lalaja, Taylor, and Caigual males had significantly more iridescent body coloration than Guanapo HP males several years after introduction. These four 2008 and 2009 introductions along with Guanapo HP are the focus here.

The goals of this study are twofold. First, we examine phenotypic convergence in tail coloration between the four experimental LP introductions in comparison with the HP ancestor from which they originated. We choose the tail as it is highly color polymorphic (Winge, 1927) and plays a key role in courtship displays (Farr, 1980), despite rarely being the exclusive subject of color studies in guppies. We hypothesize that the introduction sites will diverge from the HP site based on canopy cover and year of introduction. Second, we examine tail color gene expression differences between contemporary males from the four experimental introductions and the HP site. We hypothesize that there would be an increased expression level of color genes in all the introduction sites compared to the HP site.

## 2 METHODS

### 2.1 Study system

In 2008, juvenile guppies were removed from Guanapo HP and reared to maturity in the laboratory before being placed into mating groups. Mating groups consisted of a single tank with up to five males and five females. Additional mating groups were placed in separate tanks for a total of 38 fish of each sex. All fish were then introduced into previously guppy‐free LP reaches of the Upper and Lower Lalaja tributaries. The canopy cover was regularly thinned in Upper Lalaja to mimic that experienced by HP guppies, but left intact in Lower Lalaja. Males from a cross were introduced into a separate site from the females they mated with in the laboratory. This ensured that starting genetic diversity was similar because all males were represented in both sites, either as stored sperm in introduced females or by being themselves introduced. In 2009, a similar experiment was performed in the Taylor (thinned canopy; 52 males and 52 females) and Caigual (intact canopy; 64 males and 64 females) tributaries. All introduced fish were individually marked and photographed. A monthly mark–recapture census was initiated immediately after introduction. Virtually all guppies at a site were collected and photographed plus new recruits were individually marked, measured, and photographed, and then, all fish were released at the site of capture. After a male reached maturity (at least 14 mm in length), a photograph was taken in which the medial fins were spread open.

### 2.2 Phenotypic analysis of color convergence

**Figure 1:**
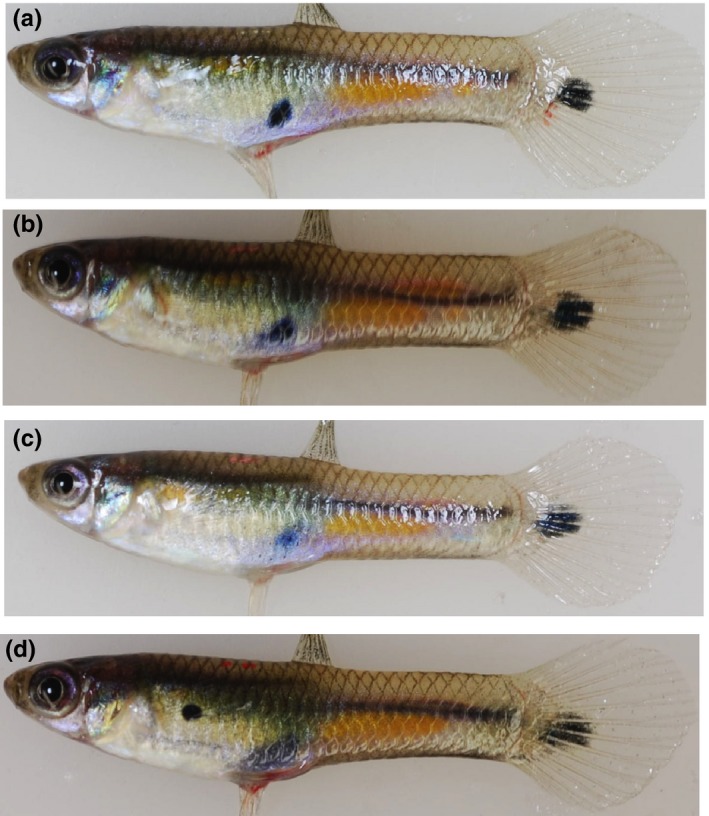
Photographs of fish described as related and unrelated. Photographs (a‐c) are males that were considered brothers based on nearly identical body and tail color elements. The male in Photograph (d) is considered unrelated to males (a‐c) as the main black body spot was in a unique location. Brothers were filtered out such that only males (a) and (d) would have been included in the analysis. All males are from Taylor and have a black spot on the front margin of the tail [Colour figure can be viewed at http://wileyonlinelibrary.com]

**Figure 2:**
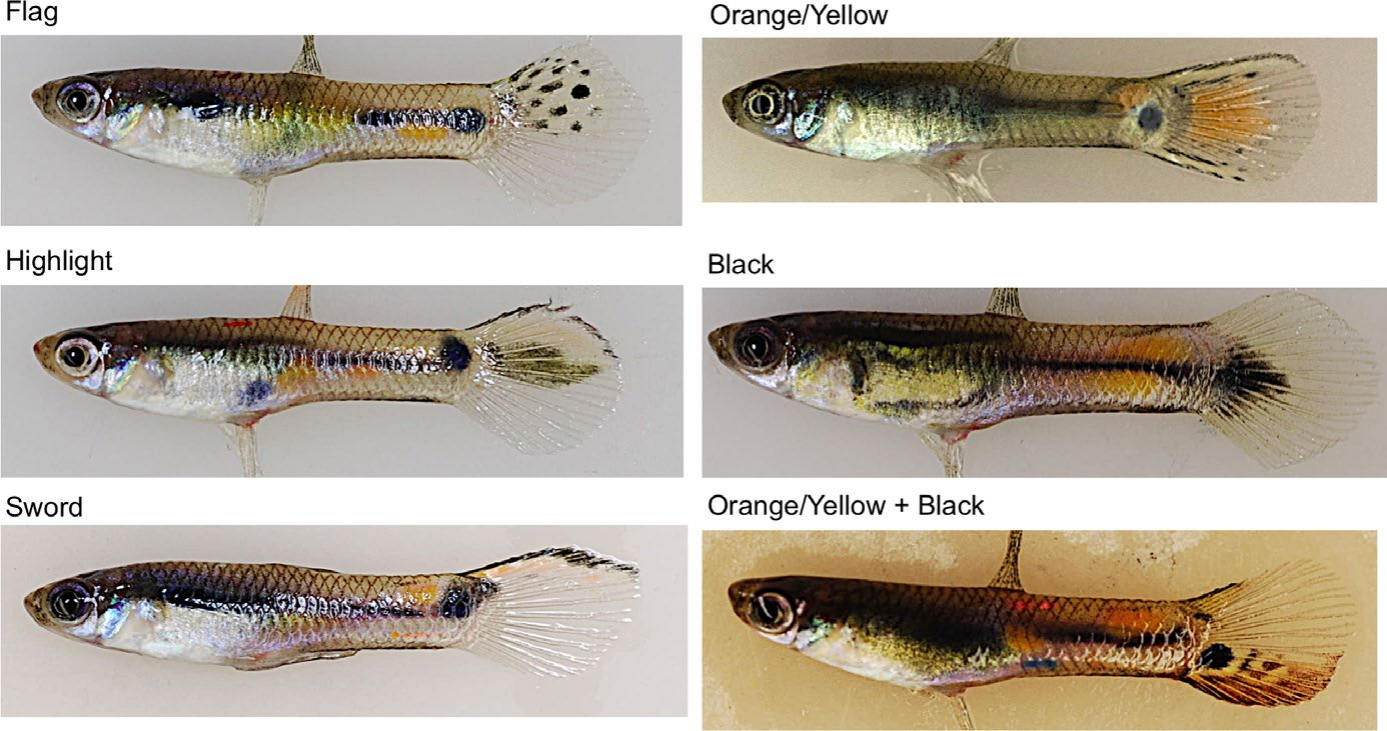
Example photographs of the color pattern categories that were scored. Note the orange/yellow fish photograph also has upper and lower swords, as that color element was never present by itself. Pattern elements could be found individually or in combination with additional elements [Colour figure can be viewed at http://wileyonlinelibrary.com]

Photographs were taken of wild‐caught adult males from the ancestral HP site located in the Guanapo River (*n* = 28 photographs) in May 2008 and from the four introduced, experimental sites of guppies four years after introduction (Upper Lalaja: *n* = 25, Lower Lalaja: *n* = 28, Taylor: *n* = 24, and Caigual: *n* = 24 photographs). All photographs analyzed have been uploaded to FigShare (https://doi.org/10.6084/m9.figshare.6130352). Potential brothers, identified based on having near identical body and tail coloration, were first filtered out such that males with identical tail color patterns within sites would not be full siblings (Figure 1). Males were visually placed into categories according to whether the tail coloration was minimal (<25% colored) or moderate (>25% colored). The tail was partitioned into three sections: proximal near the caudal peduncle, dorsal, and ventral. The distal end was not included as a partition because any distal coloration, if present, was an extension of color from another section. Sections were scored for the presence of three color patterns: flags, swords, and highlights (Figure 2). Flags consist of black spots on a field of orange/yellow. Swords are very thin stripes of black or black plus orange/yellow on the dorsal and/or ventral margins. Highlights are thick stripes of orange/yellow color bounded by black on at least two sides. If the color was present and did not conform to one of the three patterns (e.g., a single spot), it was scored as having black only, orange/yellow only, or black plus orange/yellow (Figure 2). The same researcher performed two independent categorizations for each male photograph on different days to ensure consistency. Any photographs with color score disagreements were checked a third time to correct any data entry mistakes.

To test for color pattern convergence, a correspondence analysis was performed using R package FactoMineR (Le, Josse, & Husson, 2008) on a count table of the number of individuals having 35 unique pattern types for each of the five sites. The first three axes accounted for 84.6% of the variation and were chosen for further analyses. R package factoextra was used to construct symmetric biplots plotting all five sites from the correspondence analysis.

### 2.3 Genetic analysis of color convergence

#### 2.3.1 Genetic sampling and RNA extractions

Twenty guppies were collected from Guanapo HP and the four introduction sites in 2013. Fish were bred in the laboratory at the University of California, Riverside (UCR), for two generations and maintained in a stock tank. The UCR Institutional Animal Care and Use Committee (IACUC) approved all procedures involving the fish. Male tails were sampled at a stage in the development of the anal fin when color was just beginning to develop. Fish were anesthetized in MS‐222, and 10 tails largely represented by unrelated males were combined into a single sample per site. Some of the stock males within a sample may have been brothers based on color pattern similarity, but no more than two brothers were allowed per family in a sample. This was repeated for another biological replicate taken from the stock tanks and at all five sites/stock tanks for a total of 10 samples. Fish were then immediately sacrificed in a lethal dose of MS‐222 and were not allowed to awake from anesthesia in between. Samples were flash‐frozen in liquid nitrogen and stored at −80°C until RNA extraction. RNA was extracted by homogenizing caudal fins in TRIzol (Invitrogen, Carlsbad, CA), purifying with a Qiagen RNeasy Mini Kit (Valencia, CA), and treating with TURBO DNase (Ambion, Carlsbad, CA). RNA quality was gauged with an Agilent Bioanalyzer (Santa Clara, CA). All samples had RNA integrity values ≥8.1.

#### 2.3.2 Illumina sequencing and quality control

The University of California, San Diego Institute for Genomic Medicine, constructed libraries using the Illumina TruSeq Kit v2 (San Diego, CA), following the manufacturer's recommendations and with each sample receiving a unique barcode. Samples were pooled into equimolar amounts and sequenced on two lanes of an Illumina HiSeq2500 at UCR (1 × 100 bps). The reads were cleaned using the fastq_quality_filter tool of the FASTX‐Toolkit (Hannon Lab, Cold Spring Harbor Laboratory, NY). Reads needed to have a Phred +33 quality score of at least 20 in 100% of bases to pass quality controls. Any retained reads then had residual adapter sequences trimmed using Trimmomatic 0.20 (Bolger, Lohse, & Usadel, 2014). Each sample yielded 18.8–40.8 million reads (Table 1). Raw sequence reads have been deposited in NCBI's Sequence Read Archive (Accession: SRP114275). After cleaning, there were 15.9–32.6 million reads per sample (Table 1).

**Table 1:** Number of RNA sequencing reads obtained for each sample. Cleaned reads represent the number remaining after quality control. Reads were then mapped to the annotated guppy genome

Site	Replicate	Raw reads	Cleaned reads	Mapped reads (%)
Guanapo HP	1	26,870,174	22,586,691	20,766,878 (92.0)
Guanapo HP	2	39,086,645	32,622,487	30,141,929 (92.4)
Upper Lalaja	1	21,814,280	18,212,053	16,555,056 (90.9)
Upper Lalaja	2	18,881,969	15,857,847	14,703,350 (92.7)
Lower Lalaja	1	31,063,239	26,112,166	24,316,619 (93.1)
Lower Lalaja	2	23,971,652	20,331,650	18,682,796 (91.9)
Taylor	1	32,035,905	25,681,736	23,205,491 (90.4)
Taylor	2	25,701,105	20,676,206	18,621,562 (90.1)
Caigual	1	24,075,451	19,278,165	17,624,322 (91.4)
Caigual	2	40,809,576	32,639,238	29,653,795 (90.9)

#### 2.3.3 Read alignment, read counting and differential expression

Reads passing all quality control filters were aligned in TopHat2 to the reference guppy genome (NCBI accession GCF_000633615.1) using default options, except the number of threads was four and the minimum intron size was 50 bps. Read counts for each gene were obtained using htseq‐count in union mode (Anders et al. 2010). Differential expression (DE) analyses were performed using DESeq2 (Anders and Huber 2015). A gene was only analyzed if it had at least one count per million mapped reads for at least two samples. Contrasts were generated between Guanapo HP, and each of the four introduction sites and differential expression tests utilized a false discovery rate cutoff of 0.05. Genes known to be involved in coloration (Supporting Information Table S1) were extracted from the list of differentially expressed genes.

## 3 RESULTS

### 3.1 Phenotypic analysis of color convergence

**Figure 3:**
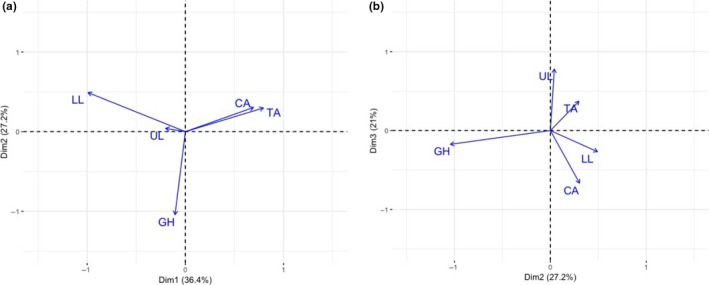
Correspondence analysis plots of principal coordinates for Guanapo HP (GH) and each of the four LP introduction sites (Upper Lalaja: UL, Lower Lalaja: LL, Taylor: TA, and Caigual: CA). (a) Dimension 1 plotted against Dimension 2. (b) Dimension 2 plotted against Dimension 3. UL and LL were founded in 2008, while CA and TA were founded in 2009. UL and TA have thinned canopies, while LL and CA have intact canopies. Acute angles between any of the sites indicate greater similarity [Colour figure can be viewed at http://wileyonlinelibrary.com]

The variation in tail color patterns among the four LP introduction sites compared to Guanapo HP was due to a mixture of nonsimilar and similar trajectories (Figure 3). The first dimension, accounting for over 36% of the variation, separated both the 2009 introductions (Taylor and Caigual) from all other sites (Figure 3a). The second dimension separated Guanapo HP from all of the introduction sites, with no trend to the separation within introductions (Figure 3a,b). The third dimension separated introduction sites according to whether their canopies were thinned (Upper Lalaja and Taylor) or intact (Lower Lalaja and Caigual) (Figure 3b).

### 3.2 Genetic analysis of color convergence

3.2.1

There are 26,071 genes in the guppy genome and 19,897 met the cutoff imposed for DE estimates of at least one count per million (CPM) in at least two samples. Of the 106 genes with at least one function in coloration located in the guppy genome, 100 met the cutoff for DE tests. There were very few DE genes (range: 77–207) between Guanapo HP and any of the individual introduction sites across the transcriptome. None of the DE genes had known functions in coloration.

## 4 DISCUSSION

Organism responses to environmental change can occur along similar or different trajectories for both phenotypes and genotypes. Identical phenotypic trajectories would be an example of convergent evolution, while varied trajectories indicate factors such as historical contingency (Achaz et al., 2014; Gould, 2002), phenotypic plasticity (Oke et al., 2016), or genetic drift (Bock et al., 2015). Phenotypic convergence with identical genetic trajectories could indicate that the phenotypes are constrained to evolve along certain directions (Schluter, 1996), while a lack of genetic convergence could indicate that the same phenotype can be produced using many‐to‐one mapping (Rosenblum, Rompler, Schoneberg, & Hoekstra, 2010).

The identification of convergence can depend on the scale being studied, and this is true for both phenotypes and genetics (general versus specific phenotypic traits or genomic regions versus base pair substitutions). In *Drosophila*, overall wing size clines were replicated across three continents, yet the exact wing features that evolved were dissimilar (Gilchrist, Huey, Balanya, Pascual, & Serra, 2004). In the same way, White Sands lizards evolved reduced pigmentation via mutations in the gene *Mc1r,* yet the molecular mechanisms were different across the species tested (Rosenblum et al., 2010). The present study has found that the biological level under examination is important when deciding to label a trait as convergent as only phenotypes were similar.

### 4.1 Phenotypic analysis of color convergence

Trait evolution after movement to a new environment can occur via selection or founder effects/drift, among other factors. Although guppy introductions were initiated with <100 individuals, we believe that congruent phenotypic changes among identical predator communities and canopy treatments are more likely the result of selection as opposed to drift.

Most of the color pattern variation explained in the correspondence analysis largely implied a lack of convergence (Figure 3a). However, there was a partial signal for the timing of introduction to be important as both the 2009 introductions (Taylor and Caigual) grouped together. For these two sites, the direction of color pattern evolution could have been biased by the starting genetic variation present. The evolution of orange pigmentation is known to evolve in parallel with female preference for orange, although the outcome of this evolution differs among guppy sites (Houde & Endler, 1990). The breeding design homogenized the starting genetic variation in males within each year of introduction, so sites introduced in the same year would be expected to have similar genetic variation for male orange ornamentation and female preference for that color. It is unclear why the 2008 introductions would not have grouped together if this were the case, although Upper Lalaja had the fewest number of color patterns present (data not shown).

The second largest percentage of variation indicated that all introduction sites had divergent color patterns from Guanapo HP, but there was no grouping trend among the introduction sites (Figure 3). Color patterns in the introduction sites were different from their HP ancestor, but they changed along unique trajectories. The divergence from Guanapo HP may be due to the selective effects of a release from predation pressure. In LP sites, sexual selection via female choice is expected to predominate (Endler, 1980) and could cause introduction sites to diverge from their HP ancestor. The unique trajectories among introduction sites could be due to historical contingency. Alternately, a lack of convergence could have occurred if there was a differential loss of color patterns among introduction sites as a result of genetic drift (Bock et al., 2015). Differences in the magnitude or direction of phenotypic differences among sites have also been found in classic systems of convergence such as stickleback, White Sands lizards, and lake whitefish (Kaeuffer, Peichel, Bolnick, & Hendry, 2012; Rosenblum & Harmon, 2010; Siwertsson, Knudsen, Adams, Praebel, & Amundsen, 2013).

The third largest percentage of variation implied convergent evolution as that dimension separated sites according to canopy cover (Figure 3b). This change to color pattern is significant, as it happened regardless of any biases to the starting genetic variation present at introduction. Light environment must play a more important role in the direction of evolution. Another recent study determined that canopy cover impacted guppy body coloration, with the greater divergence between Guanapo HP and the thinned canopy sites (Kemp et al., in review). Canopy cover could impact the direction of pattern evolution in one of two ways: First, additional light increases food availability via increased primary productivity, which leads to stronger orange/yellow carotenoid colors (Grether, Hudon, & Millie, 1999; Grether, Millie, Bryant, Reznick, & Mayea, 2001); and second, the light visibility could impact color pattern viewing. Variable light environments can cause the differential evolution of sexual signals through sensory drive (Boughman, 2002; Endler & Basolo, 1998). If female neural capacity was altered under different canopies and this caused a shift in female preferences, certain colors or patterns might be favored. Rivers with thinner canopies are brighter in all wavelengths and are enhanced for shorter (blue‐UV) wavelengths in shaded areas, while rivers with thicker canopies are enhanced for middle‐to‐long wavelengths (Endler, 1993). As a result, different color patterns may be favored to increase visibility and color contrast.

### 4.2 Genetic analysis of color convergence

4.2.1

Phenotypic color pattern evolution of the introduction sites was not matched with genetic convergence in color gene expression. The statistical lack of DE color genes between Guanapo HP and the four introduction sites could be caused by a couple of explanations. First, there may not yet be a strong expression component to coloration in these sites. Gordon et al. (2015) determined in a common garden study that alterations in black coloration were a plastic response to the environment. The fish reared in the present study were bred in the laboratory for two generations to reduce environmental effects so changes to black coloration may not have been detectable in a genetic analysis. Gordon did find that orange/yellow coloration had a genetic component in the common garden, but such genetic changes were not detected here. A previous study (Dick, Arendt, Reznick, & Hayashi, in review) found that genes underlying orange/yellow coloration are most differentially expressed at an earlier developmental stage (when there is no visible color), so it is possible that the fish stage sampled here (developing coloration) was too advanced to detect gene expression differences in that coloration.

Second, the sampling protocol used for genetic analyses may have been inadequate to quantify gene expression changes to coloration. Due to logistical constraints, samples consisted of a pool of tails from 10 males taken in a large stock tank. It is difficult to obtain a sample of 10 that represents an entire site when sites may have 1–2 orders of magnitude more fish censused in any given month. The biological replicates at a site were not always similar based on MDS plots (not shown) created during expression analysis, which also supports the idea that the sampling protocol had inadequate density.

Third, there were very few differentially expressed genes identified between HP and LP fish across the whole transcriptome. This would indicate that genetic differences between HP and LP male tails are minimal or could be reflected in changes other than gene expression, such as sequence variation. Alternatively, pooling largely unrelated males within samples may have caused individual significant differences in gene expression to go undetected if those differences were not shared with the other males in the pool.

## CONFLICT OF INTEREST

None declared.

## AUTHOR CONTRIBUTIONS

CD, CYH, and DNR conceived the study. CD and JH collected tail samples and analyzed the genetic data. CD performed RNA extractions and analyzed the phenotypic data. All authors contributed to manuscript writing.

## DATA ACCESSIBILITY

RNA‐seq data files are available at the Sequence Read Archive (SRA) under Accession SRP114275. Photographs are uploaded to FigShare (https://doi.org/10.6084/m9.figshare.6130352).
